# A survey of protocols and trends in orthodontic retention

**DOI:** 10.1186/s40510-017-0185-x

**Published:** 2017-10-09

**Authors:** Alvyda Andriekute, Arunas Vasiliauskas, Antanas Sidlauskas

**Affiliations:** Clinic of Orthodontics, Lithuanian University of Health Sciences, Lukšos-Daumanto st. 6, LT-5016 Kaunas, Lithuania

**Keywords:** Retention, Hawley retainer, Fixed retainer

## Abstract

**Background:**

The objectives of this study were to evaluate retention procedures and protocols which are used by the orthodontists in Lithuania and to identify commonly used types of dental retainers.

**Methods:**

One hundred seven questionnaires in total with 28 multiple-choice questions were sent to all members of the Lithuanian Orthodontic Society. The questionnaire was organized into eight sections representing specific information about socio-demographic status of the respondents, selection of a retention system, details of commonly used fixed and removable retainers, the duration of the retention period, supervision of the retainers, instructions for patients, and necessity of common retention guidelines.

**Results:**

The overall response rate was 75.7%. All of the respondents prescribed retainers after the orthodontic therapy. More than 40% of the respondents combined fixed and removable retainers in different clinical situations, but the first-choice option after an expansion of the maxillary dental arch was the removable retainer (54.3%); meanwhile, a fixed retainer was used after a correction of any rotations of the mandibular anterior teeth (49.4%). The Hawley retainer was preferred by 90.1% of the respondents for a maxillary dental arch, and 74.1% of them preferred it for a mandibular dental arch. The most preferable fixed retainer was the retainer bonded to all six anterior teeth (in the upper dental arch—by 71.6%; in the lower one—by 80.2%). There was no consensus on the duration of a retention period. Most of the orthodontists checked up retainers three times during the first year (fixed ones—by 42.0%; removable ones—by 30.0%) and once per year after the 1-year retention period (fixed ones—by 44.4%; removable ones—by 40.7%). All orthodontists gave instructions for taking care of an orthodontic retainer. It was observed that the orthodontists with less than 10 years of experience used a protocol based on the skills learned during their postgraduate studies, while orthodontists with more than 10 years of experience used retention procedures based on their orthodontic work practice (*p* < 0.05).

**Conclusions:**

A combination of fixed and removable retainers was the most often used in an orthodontic retention. Evidence-based guidelines are desired for a common retention protocol.

## Background

There is no doubt that teeth after an active orthodontic treatment have a tendency to move into the previous position, and a relapse can occur at any age [1]. The supragingival and transseptal fibers are most commonly associated with a relapse; occlusal factors, soft tissue pressures, and further growth are also some influencing factors [2]. A relapse affects patients’ time and finances and can cause esthetic discomfort because unfavorable changes often appear in the front teeth. This situation negatively affects both the patient and the doctor. Orthodontic retainers which are made to be worn after dental braces in order to maintain teeth in their correct position are used to minimize any relapse.

Nevertheless, there is no agreement among the orthodontists concerning the need for any retention, choosing the type of a retainer, or determining how long retainers should be worn after an orthodontic treatment. A large number of variations in retention strategies, different materials for retention, or individual patient factors can lead to challenges of choosing retention protocols. Orthodontic materials and methods are constantly changing and manufacturers suggesting new alternatives. Despite the fact that a growing number of surveys of protocols and trends in orthodontic retention that have been conducted in different countries have revealed some tendencies between the orthodontists [3–11], further studies are needed for the development of a retention protocol. The common retention protocol is an attempt to systemize and standardize retention procedures which would be useful for orthodontists. Meanwhile, no research has been accomplished on the most often used dental retention system among Lithuanian orthodontists. The main purposes of this study were to evaluate the protocols and trends used in an orthodontic practice and to identify any commonly used types of dental retainers.

## Methods

The survey questionnaire was developed according to similar studies [8, 9] and edited and prepared in the Lithuanian language. The questionnaire consisted of 28 multiple-choice questions, and it was divided into eight parts representing some specific information. It was possible to select one or even multiple answers from the list of options.

### Socio-demographic status of the respondents

Firstly, there was a socio-demographic status of the respondents included, and they were asked to identify their gender, university where they have completed their postgraduate studies, the work sector, and length of their independent work as an orthodontist after having finished postgraduate studies.

### Selection of a retention system

The second section examined if orthodontists used retention appliances after orthodontic treatment, which types of retainers were typically used for applied treatment, conditions or malocclusions (for patients with anterior open bite, impacted anterior teeth, etc.), and factors influencing their selection of the retainer type.

### Fixed retainers

Part 3 referred to the most often used fixed retainers and the details of commonly used fixed retainers (material, type, form, and diameter) and examined which teeth were used for bonding in the upper and lower dental arches and the methods and contraindications of bonding a fixed retainer in finished cases.

### Removable retainers

The fourth section gathered information about the most often used removable retainers (Hawley retainer, Begg retainer, clear (vacuum-formed) retainer, etc.) for the upper and lower dental arches.

### The duration of the retention period

The fifth section consisted of questions about a retention period—respondents were asked to note the duration of the primary retention, prescription of wearing a removable retainer during and after the primary retention period, and details of wearing a fixed retainer.

### Supervision of the retainers

Part 6 was dedicated to question who is responsible for patients in retention and information on the number of any follow-up visits per year after the prescription of fixed or removable retainers.

### Instructions for patients

The seventh part requested information if orthodontists gave any instructions for the patient/patient’s parents (written or verbal) about the maintenance of removable or fixed retainers, which instructions are provided after the bonding of a fixed retainer, which oral hygiene measures are recommended for fixed retainers, and what are the recommendations in case there are some issues with retainer. Also, we gathered information if general practitioners are involved in the maintenance of fixed retainers and what are the recommendations if a dentist during the examination has noticed a disengaged/broken retainer.

### The necessity of common retention guidelines

Finally, respondents were asked to specify the reasons for using a retention protocol and if they felt the necessity of a general retention procedure protocol.

A questionnaire study was conducted from January to March 2016 by handing out anonymous paper questionnaires to orthodontists or sending electronic ones. The names and email addresses of the orthodontists were collected from the Lithuanian Association of Orthodontists. Email remainders were sent a week later to those who have not completed or partially completed the survey. This action was repeated 2 weeks later. All members of the Lithuanian Orthodontic Society were included in this survey.

A statistical analysis was performed by collecting data and analyzing with the software package SPSS 21.0. The data were expressed as a frequency and percentage. Significance between differences was evaluated by a chi-square test. The *p* value of < 0.05 was considered as statistically significant. Binary logistic regression analyses were carried out to identify any factors associated with the choice of a retention according to the length of the work experience.

## Results

### Socio-demographic status of the respondent

One hundred seven questionnaires were sent out, and 81 were returned completed. The attained response rate was 75.7%. Altogether, 72 orthodontists and 9 postgraduate students returned the submitted surveys: 86.4% of them were females and 13.6% were males. The majority of the respondents (55.6%) were working only in the full-time private practice sector, 19.8% mentioned a combination of the public and the private practice sectors, 2.5% worked at the university, and 1.2% were retired (partly). Another 14.8% of the respondents worked in the private practice sector, at the university, and in the public clinic, while 6.2% of the orthodontists noted the university and the public clinic as their workplaces. A total of 74 orthodontists were trained in Lithuania (56.8% at the Lithuanian University of Health Sciences, 43.2% at the Vilnius University), and the remaining 7 orthodontists were trained in other countries. The orthodontists were asked to specify their work experience: 38 (46.9%) respondents had less than 10 years of experience in the treatment of orthodontic patients, while 43 (53.1%) orthodontists had more than 10 years of working experience.

### Selection of a retention system

**Fig. 1:**
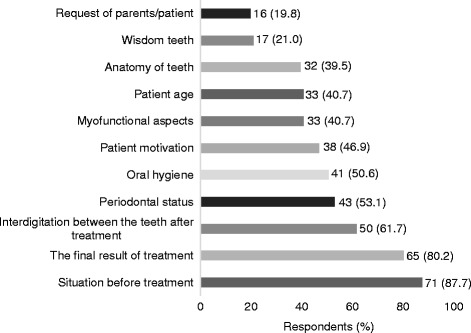
Absolute numbers and percentage of the respondents who noted the factors influencing their choice of retention

All respondents prescribed retainers after the orthodontic treatment—bonded, removable, or both. A total of 87.7% of the respondents to the question “What are the main factors influencing the choice of a retention?” answered that the main factor was the patient’s dental condition before the treatment. The final result of the treatment, interdigitation between the teeth, patient’s oral hygiene, and periodontal tissue status were mentioned by the majority of the orthodontists as the factors affecting orthodontists’ choice of the retention (Fig. 1).

A combination of a bonded and a removable retainer was mostly used by the orthodontists in both dental arches, except after expansion of the upper dental arch when a removable retainer was dominant, and after the correction of a rotation of the permanent mandibular anterior teeth, the orthodontists preferred a fixed retainer (Tables 1 and 2).

Three respondents (3.7%) used only removable retainers, whereas 17 respondents (21.0%) used both fixed and removable retainers in all cases.

### Fixed retainers

The most preferred fixed retainer was the retainer bonded to all anterior teeth (canine to canine). 80.2% of respondents bonded a fixed retainer in the lower jaw to all six anterior teeth, and 71.6% of them did it in the upper jaw (Table 3).

**Table 1:** Respondents who chose a certain retention in the upper dental arch depending on the condition or treatment

Condition/treatment	Fixed	Removable	Both	None
Extractions	1 (1.2)	29 (35.8)	51 (63.0)	–
Diastema closure	22 (27.2)	7 (8.6)	52 (64.2)	–
Expansion of the dental arch	1 (1.2)	44 (54.3)	36 (44.4)	–
Crowding in the anterior teeth	9 (11.1)	20 (24.7)	52 (64.2)	–
Impacted anterior teeth	9 (11.1)	24 (29.6)	48 (59.3)	–
Intrusion of the anterior teeth	8 (9.9)	29 (35.8)	43 (53.1)	1 (1.2)
Extrusion of the anterior teeth	13 (16.0)	21 (25.9)	46 (56.8)	1 (1.2)
Rotations of the anterior teeth	19 (23.5)	21 (25.9)	41 (50.6)	–
Anterior open bite	4 (4.9)	31 (38.3)	46 (56.8)	–
Retaining overjet (OJ)	4 (4.9)	35 (43.2)	42 (51.9)	–
Root resorption of the anterior teeth	15 (18.5)	26 (32.1)	36 (44.4)	4 (4.9)
Previous orthodontic treatment	4 (4.9)	24 (29.6)	53 (65.4)	–
Adult patient	7 (8.6)	21 (25.9)	53 (65.4)	–

Values are presented as numbers (%)

**Table 2:** Respondents who chose a certain retention in the lower dental arch depending on the condition or treatment

Condition/treatment	Fixed	Removable	Both	None
Extractions	19 (23.5)	11 (13.6)	51 (63.0)	–
Diastema closure	34 (42.0)	3 (3.7)	41 (50.6)	3 (3.7)
Expansion of the dental arch	12 (14.8)	21 (25.9)	47 (58.0)	1 (1.2)
Crowding in the anterior teeth	35 (43.2)	3 (3.7)	43 (53.1)	–
Impacted anterior teeth	28 (34.6)	8 (9.9)	44 (54.3)	1 (1.2)
Intrusion of the anterior teeth	27 (33.3)	10 (12.3)	42 (51.9)	2 (2.5)
Extrusion of the anterior teeth	27 (33.3)	10 (12.3)	42 (51.9)	2 (2.5)
Rotations of the anterior teeth	40 (49.4)	8 (9.9)	33 (40.7)	–
Anterior open bite	24 (29.6)	14 (17.3)	43 (53.1)	–
Retaining overjet (OJ)	18 (22.2)	15 (18.5)	48 (59.3)	–
Root resorption of the anterior teeth	28 (34.6)	18 (22.2)	33 (40.7)	2 (2.5)
Previous orthodontic treatment	20 (24.7)	9 (11.1)	52 (64.2)	–
Adult patient	21 (25.9)	9 (11.1)	51 (63.0)	–

Values are presented as numbers (%)

**Table 3:** Respondents who chose a certain bonding type of fixed retainers

Bonding types of fixed retainers	Maxillary retainer	Mandibular retainer
Bonded to the canines only	3 (3.7)	7 (8.6)
Bonded to all anterior teeth	58 (71.6)	65 (80.2)
Bonded to central incisors	18 (22.2)	2 (2.5)
Bonded to all incisor teeth	29 (35.8)	7 (8.6)
Bonded to all teeth from the first premolar to the first premolar	11 (13.6)	23 (28.4)

Values are presented as numbers (%)

The majority of the orthodontists (76.5%) adjusted the arch wire retainers clinically. While choosing the material for a fixed orthodontic retainer, 71.6% of the respondents noted a stainless steel wire. Braided wire was chosen by 75.3% of the specialists, and the dominant form was a rectangular one (66.7%).

**Fig. 2:**
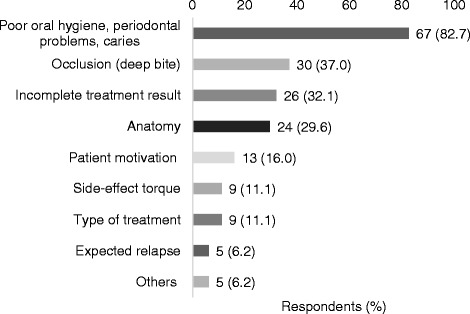
Absolute numbers and percentage of the respondents who mentioned the contraindications for using fixed retainers

The most frequently mentioned contraindications for fixed retainers were caries, a periodontal disease, poor oral hygiene, deep bite, incomplete treatment result, and the anatomical characteristics of the teeth (Fig. 2).

### Removable retainers

The most popular removable retainers in the upper and lower dental arches were the Hawley and the vacuum-formed retainers (Table 4).

**Table 4:** Respondents who used removable retainers

Removable retainers	Upper arch	Lower arch
Hawley retainer	73 (90.1)	60 (74.1)
Vacuum-formed retainer	62 (76.5)	59 (72.8)
Functional appliances	43 (53.1)	38 (46.9)
Positioner retainer	23 (28.4)	
Begg retainer	16 (19.8)	10 (12.3)
The Damon Splint	5 (6.2)	
Spring retainer	3 (3.7)	5 (6.2)
External distraction devices	2 (2.5)	2 (2.5)
Others	1 (1.2)	1 (1.2)

Values are presented as numbers (%)

### The duration of the retention period

The duration of the retention period was from 1 month to the entire life, and 1 year was the most frequently mentioned as the first retention period by the orthodontists (30.9%). The patients during this period should wear their removable retainers for a certain amount of time, and the range of this time varied from 6 to 24 h. The most often mentioned answers were 24 h per day/7 days per week (29.6% of the respondents). Thirty seven percent of the orthodontists in total noted that the removable retainers should be worn for 5 years and more after the completion of an active tooth movement, while others (34.6%) recommended to wear removable retainers for 1–2 years. A vast majority of the respondents (92.6%) did not remove any fixed retainers after the first retention period unless the treatment of anterior teeth was planned (49.4%) or the patient had a poor oral hygiene (39.5%).

### Supervision of the retainers

The answers to the question “Who is responsible for the regular inspections during retention period?” were divided into three camps: 53.1% of the respondents indicated that the orthodontists are responsible for the regular inspections of retainers, whereas 40.7%, the patients/their parents. The remaining respondents noted general dentists as an option. The orthodontists recommended three appointments during the first year for inspection of retainers (fixed ones—by 42.0%; removable ones—by 30.0%) and once per year after the 1-year retention period (fixed ones—by 44.4%; removable ones—by 40.7%).

### Instructions for patients

All orthodontists gave instructions for retainers. These instructions included information about any detachment and breakage of the retainers (95.1%), nutrition (66.4%), follow-up visits (87.7%), and oral hygiene (93.8%). The patients wearing removable retainers were given oral (63.0%), written (32.1%), or oral/written (4.9%) information, and the patients with fixed retainers were informed orally (66.7%), in writing (29.6%), or in both ways (3.7%). Oral hygiene tools such as a toothbrush, dental floss, mouthwash, interdental brushes, and oral irrigators were recommended by more than 50% of the respondents.

More than 90% of the respondents recommended to inform the orthodontist in case of problems that can arise with all types of retainers. 48.1% of the orthodontists in total communicated with general dentists regarding any inspection and repair of the retainers. The orthodontists (74.1%) noted that general dentists should refer the patients to the orthodontists if the fixed retainer has broken or became loose.

### The necessity of common retention guidelines

The participating orthodontists were asked to identify factors influencing the decision for the retention protocol in use. The results of a binary logistic regression analysis showed that two factors influencing the decision for the retention protocol in use were associated with the work experience in orthodontics. Younger orthodontists with less than 10 years of experience were 3.85 more likely (95% CI 1.40–10.63) to choose “Knowledge and skills gained in orthodontic residency” as compared to those orthodontists with more than 10 years of experience. The orthodontists working more than 10 years in practice were 7.78 more likely (95% CI 2.03–29.87) to choose a “clinical experience.” The years of work experience appear to be a significant determinant for choosing the retention protocol. It can be predicated that specialists with less than 10 years of experience used a retention protocol based on the skills learned during the postgraduate studies while orthodontists with more than 10 years of experience used a retention protocol based on the orthodontic work practice (*p* < 0.05) (Table 5).

**Table 5:** The orthodontists who used a retention protocol based on factors according to the length of their work experience

Factors influencing the choice of a retention protocol	Number	Work experience (years)		*p*
		≤ 10 years (*n* = 38)	> 10 years (*n* = 43)	
Knowledge and skills gained in postgraduate studies	81	31 (81.6)	23 (53.5)	0.007*
Clinical experience	81	24 (63.2)	40 (93.0)	0.001*
Knowledge gained from orthodontic books	81	18 (47.4)	26 (60.5)	0.238
Knowledge gained from the Internet	81	17 (44.7)	14 (32.6)	0.260
Other factors	81	6 (15.8)	3 (7.0)	0.208

Values are presented as numbers (%) by a chi-square test

*p < 0.05

A common retention protocol would be helpful; such an opinion was prioritized by the orthodontists in Lithuania (98.7%).

## Discussion

There are currently many different types of removable and fixed retainers, and it is unclear which retainers are the best and how long they should be used [12]. This study investigated the existing retention protocols used by the orthodontists in Lithuania. A survey involving all 98 licensed Lithuanian orthodontists was conducted, and the obtained data represented the opinions of the specialists on the retention procedures. Nine postgraduate students were also included in the survey, thus demonstrating that their opinions as ones of future professionals are significant, although the inclusion of postgraduate students might not be that straightforward, because normally, they use the retention protocols of the clinical instructors and are not free to develop their own choice based on clinical experience. The response rate of 75.7% was relatively high compared with the surveys conducted in the other countries [3–11]. It showed that this study was relevant to the interests of the orthodontists. On the other hand, some orthodontists noted that the questionnaire was too long and it took a lot of time.

Previous surveys conducted in certain countries have raised the main questions related to the selection of a retainer and the duration for wearing a retainer. Although the orthodontists chose different retainers for different orthodontic situations, some trends were observed. Surveys performed in the other European countries [4, 5, 8, 9], USA [7], Saudi Arabia [11], and Australia and New Zealand [3] showed that fixed retainers for a lower dental arch were dominant, except in Ireland [6] and Malaysia [10], where vacuum-formed retainers were the most popular choice. The opinions regarding an orthodontic retention in the upper dental arch were various: fixed retainers were most often chosen in Switzerland [9] and the Netherlands [4], Hawley retainers in the USA [7] and Saudi Arabia [11], and vacuum-formed retainers in the UK [5], Ireland [6], and Malaysia [10]. A combination of a fixed and removable retainer (a vacuum-formed retainer) was the most commonly used in Norway [8], and this was in agreement with our study; however, the orthodontists in Lithuania gave priority to the Hawley retainers. Lithuanian orthodontists preferred a combination of a fixed and removable retainer in the upper and lower arches, except after an expansion of the maxillary dental arch or correcting any rotations of the mandibular anterior teeth. The reason for a “double” retention might be that the orthodontists were worried about the relapse tendency and about the patients who might forget to wear their removable retainer as specified. Additionally, the findings of the study by Atack et al. [13] showed similar results between fixed and removable retainers and confirmed that a relapse in the lower front teeth can occur with both types of retainers.

More than 70% of the orthodontists in Lithuania preferred retainers to be fixed to all six anterior teeth, and this way of fixation was dominant in upper and lower arches. In that aspect, our results were in line with a study conducted by Keim et al. [14], which showed that fixed retainers bonded to all anterior teeth (3–3) particularly in the mandibular arch which were in the ascendant. Orthodontic canine-to-canine retainers were considered to be effective [15] and invisible [16] and could ensure permanent retention and alignment of the anterior teeth [17, 18]. Other advantages were mentioned by the researchers: good patient acceptance [16] and low failure rate [16, 17]. Nevertheless, fixed retainers could cause difficulties for patients to reach areas with a toothbrush or a dental floss, increase plaque accumulation, and influence the periodontal health [19]. However, another study showed that fixed retainers allow patients to maintain good hygiene and periodontal status [17].

The frequency and the duration of wearing a retainer are still widely discussed today among orthodontists. A majority of the Lithuanian orthodontists (30.9%) considered that 1 year is the optimal time interval for the first retention period. This view was supported by the study conducted by Parker [20] which demonstrated that at least 232 days of retention are needed to ensure the regeneration of the fibers surrounding the apical, middle, and marginal areas of the root and to provide the stability after an orthodontic treatment. Destang and Kerr [21] compared the retention time in the maxillary arch and found that the 1-year retention showed a better stability of the teeth position than the one of 6 months. One year after the braces were taken off, more than 90% of Lithuanian orthodontists left the retainers bonded in place for an unlimited time. If the oral hygiene of a patient was poor and it could not be improved or a dental treatment was planned for the front teeth, the fixed retainer was removed. Similar results were obtained in other countries such as the Netherlands [4], Switzerland [9], UK [5], Ireland [6], USA [7], Malaysia [10], and Saudi Arabia [11] where orthodontists tended to leave fixed retainers indefinitely.

The orthodontists seemed to be split in our study into two camps with regard to the duration of wearing a removable retainer after an orthodontic treatment, and they recommended to wear a removable retainer for 1–2 years or 5 years and more. Some studies showed that the orthodontic treatment results are not stable in the long term and even after 10 or 20 years, the stability and good alignment of teeth are not guaranteed [22]. This confirmed the opinion that orthodontic patients should wear their retainers for life in order to maintain their stable results as long as possible [23, 24]. Mandibular dental arches became narrower and shorter over time in patients after an orthodontic treatment. The same tendency was observed in untreated patients, and it showed that this is associated with physiological processes or dental arch maturation. Natural aging processes also could affect the occlusion, and it could cause some overcrowding or changes in the dental arch dimensions [25–27]. Anyway, a long-term retention and regular checkups for any orthodontic patients are desirable because they could prevent a relapse in the lower front teeth and changes in the occlusion [24, 25, 28].

Only half of the orthodontists communicated with general dentists regarding an inspection and repair of the retainers, and it showed that there is no close connection between general dentists and orthodontists. A similar situation to that in Lithuania was observed in Switzerland [9], where 62% of the orthodontists maintained a successful relationship with general dentists. General dentists are important because they not only choose the orthodontist according to their good relationships with the general dentist, reputation, and other factors [29] but they are also involved in the treatment [30]. If appropriate, patients after an orthodontic treatment return to their general dentists in order to undertake any needed dental treatment including their oral hygiene, extractions, restorations, or implantation. This confirms that common retention protocol is desirable, and teamwork plays an important role in the treatment and the final result.

## Conclusions

A combination of fixed and removable retainers was the most often used in the orthodontic retention. The Hawley appliance was a predominant removable retainer. The bonded wire from canine to canine was the most frequent fixed retainer. Evidence-based guidelines are desired for a common retention protocol.
